# Influence of Acculturation on Risk for Gestational Diabetes Among Asian Women

**DOI:** 10.5888/pcd16.190212

**Published:** 2019-12-05

**Authors:** Liwei Chen, Lu Shi, Donglan Zhang, Shin Margaret Chao

**Affiliations:** 1 Department of Epidemiology, Fielding School of Public Health, University of California Los Angeles, Los Angeles, California; 2 Department of Public Health Sciences, Clemson University, Clemson, South Carolina; 3 Department of Health Policy and Management, University of Georgia, Athens, Georgia; 4 Department of Public Health Los Angeles County, Maternal, Child, and Adolescent Health Programs, Los Angeles, California

## Abstract

**Introduction:**

Asian women have a higher prevalence of gestational diabetes mellitus than women of other races/ethnicities. We aimed to compare the prevalence of gestational diabetes among Asian American women to other racial/ethnic groups and explore whether the higher occurrence of the disorder among Asian women can be explained by acculturation.

**Methods:**

We conducted a population-based, cross-sectional study among 5,562 women who participated in the 2007 Los Angeles Mommy and Baby Study (LAMB) in Los Angeles County, California. All women included in this study had a live delivery in 2007 and did not have pre-pregnancy type I or II diabetes. We applied multivariate, weighted logistic regressions to compare gestational diabetes prevalence among racial/ethnic groups, adjusting for its known risk factors. We conducted mediation analysis to test whether the difference in prevalence across racial/ethnic groups could be explained by acculturation.

**Results:**

Among the 5,562 women studied, the weighted prevalence of gestational diabetes was 15.5% among Asian American women, followed by 9.0% among non-Hispanic black women, 10.7% among Hispanic women, and 7.9% among non-Hispanic white women. Compared with non-Hispanic white women, Asian women had 2.44 (95% confidence interval [CI], 1.81–3.29; *P* < .001) times the odds of having gestational diabetes, independent of maternal age, education, marital status, income, prenatal care adequacy, prepregnancy BMI, and physical activity. Acculturation was negatively associated with having gestational diabetes (odds ratio [OR] = 0.93; 95% CI, 0.86–0.99) and explained 15.9% (95% CI, 11.38%–25.08%; *P* < .001) of the association between Asian race and the condition.

**Conclusion:**

We found that Asian race was an independent risk factor for gestational diabetes, and higher acculturation may play a protective role against it in Asian American women.

**Summary**

**What is already known about this topic?**

Asian women have a higher prevalence of gestational diabetes mellitus than women of other races. However, little data exist on why prevalence is highest among Asian women.

**What is added by this report?**

We conducted a population-based, cross-sectional study among 5,562 women with a live delivery in 2007 in Los Angeles County, California. We found that Asian women had 2.44 times the odds of having gestational diabetes as non-Hispanic white women. The association was independent of maternal age, education, marital status, income, prenatal care adequacy, prepregnancy body mass index, and physical activity. We also found acculturation was inversely associated with gestational diabetes and could explain 16% of the association between Asian race with gestational diabetes.

**What are the implications for public health practice?**

Gestational diabetes is one of the most common pregnancy complications in the United States. Clinicians should be aware of the high gestational diabetes risk in Asian women.

## Introduction

Gestational diabetes mellitus, one of the most common pregnancy complications, is defined as having any degree of glucose intolerance with onset of pregnancy (1). Recent data showed a significant increase globally in the prevalence of the condition among women of various ethnic/racial backgrounds and in different geographic regions (2,3). This trend is likely to continue because of the rise in obesity rates for women of reproductive age. Although most women return to normal glucose status after delivery, 20% of women with gestational diabetes develop impaired fasting glucose or impaired glucose tolerance 6 to 12 weeks postpartum (4,5). More importantly, women with gestational diabetes are at increased risk of developing type 2 diabetes later in life. This increased risk has been documented in different populations and countries. On average, the risk of developing type 2 diabetes is 7.4 times greater for women with gestational diabetes than for women without (4).

It is well documented that gestational diabetes prevalence is higher among Asian women than among non-Hispanic white, non-Hispanic black, or Hispanic women (6). Articles published over the past 20 years with either population-based studies with sample size at or above 500 or hospital-based studies with sample size at or above 1,000 in which at least 70% of the population was screened showed that although the prevalence of gestational diabetes varied worldwide, the Southeast Asia region consistently had the highest prevalence (6). Even in studies within 1 country such as the United States (7–15), the United Kingdom (16), Switzerland (17), and Australia (18), Asian women had the highest gestational diabetes rate among all racial/ethnic groups.

However, little data exist on why prevalence is highest among Asian women. Most previous studies that reported gestational diabetes prevalence among different racial/ethnic groups in the United States relied on birth certificate data (13), insurance data (14), or hospital discharge data (15), which are known to be subject to missing data (19). Moreover, earlier studies did not have detailed information on women’s lifestyle, behavioral, and psychosocial factors or social support, acculturation, and neighborhood contextual factors (14). Acculturation is the social process by which racial/ethnic minority people adopt the behaviors, practices, attitudes, and values of the host country (20). Higher acculturation was associated with increased risk of diabetes among Hispanic populations in a US study (21) and among Chinese populations in an Australian study (22). Because Asians are the largest and fastest-growing group of new immigrants to the United States, with approximately 74% foreign-born (23), it is worth exploring how socio-cultural factors such as acculturation might play a role in these associations. Such information is critical for gaining better understanding of the underlying reasons why Asian women have the highest gestational diabetes prevalence globally and within a country.

We examined gestational diabetes prevalence by race/ethnicity and by subgroups of Asian women in a population-based study in Los Angeles County, California. We also examined whether the disparities in prevalence observed was independent of known risk factors such as maternal demographics (eg, age, education, marital status, income), prepregnancy body mass index (BMI) (weight in kg divided by height in square meters), adequacy of prenatal care, physical activity, stress during pregnancy, and neighborhood contextual factors. Furthermore, we assessed whether acculturation played a role in the association between race/ethnicity and gestational diabetes prevalence.

## Methods

### Study population

Our study population was women who participated in the 2007 Los Angeles Mommy and Baby (LAMB) study. LAMB was a population-based, multilevel, cross-sectional study of women with a live delivery in 2007 in Los Angeles County. To ensure that the final LAMB sample represented the entire live birth population in Los Angeles County, as well as racial diversity and place of residence by Service Planning Areas (the 8 geographic units of Los Angeles County that the county government identifies for planning purposes), the LAMB study applied 3 sampling processes: 1) selection of neighborhoods based on census tracts, 2) selection of births within these neighborhoods, and 3) a final supplementary sample of eligible women to create a final county sample for routine surveillance purposes. Detailed information about the LAMB study design and sampling strategies was reported in a previous study (24). Los Angeles County was divided into 2 strata, high-risk and low-risk, on the basis of 6 perinatal indicators: 1) percentage of women of reproductive age with annual incomes below 200% of the federal poverty guidelines, 2) percentage of women receiving Medi-Cal (California Medicaid program) at delivery, 3) percentage of women aged 18 or younger, 4) percentage of low birthweight children, 5) percentage of women with late onset prenatal care or no prenatal care, and 6) the infant mortality rate. Overall, 300 census tracts were selected, 200 of which were from the high-risk stratum, to achieve an adequate sample of high-risk tracts. Eligible participants were recruited 4 to 7 months after a live birth. For twins or triplets, 1 baby was randomly selected. Our final study sample was 5,562 women who had no history of diabetes before the index pregnancy.

**Assessment of GDM and race/ethnicity. **Women with gestational diabetes were identified on the basis of self-reported information. In the 2007 LAMB survey, women were asked whether they had high blood glucose that started during this pregnancy (yes or no). Maternal race/ethnicity was obtained from 2007 California birth certificate data.

**LAMB survey instrument and measures**. The 2007 LAMB survey consisted of 80 prevalidated questions developed originally from the Pregnancy Risk Assessment Monitoring System. The survey assessed preconception health (eg, type of insurance, pregnancy intention, folic acid/multivitamin use, contraception use, tobacco use, parity, previous birth outcomes), pregnancy variables (eg, access to and quality of health care, pregnancy complications, maternal stress, maternal self-esteem, maternal smoking, alcohol and drug use, food insecurity, partner conflict/support, social network support, neighborhood service/support, racial discrimination), postpartum care and infant health (eg, birthweight, breastfeeding, baby sleeping pattern, well-baby and postpartum checkups), sociodemographic information of the mothers (eg, annual household income, marital status, education, occupation), and acculturation (eg, nativity, language spoken at home, length of residence in the United States). The survey was mailed to study participants with follow-up attempts and administrated by telephone interview for nonrespondents and those who asked to complete the survey by telephone, with a response rate of 35.7% (24). The LAMB survey was translated into Spanish and Mandarin Chinese, and a telephone translation service provided access for people who spoke any one of 88 languages. The LAMB 2007 study was approved by both the University of California, Los Angeles, and Los Angeles County institutional review boards.

**Assessment of acculturation score**. The LAMB survey asked women 3 questions about their acculturation status: country of birth, language spoken at home, and length of time in the United States. An acculturation score was calculated on the basis of their responses to these questions. A 0 to 3 score was assigned on the basis of country of birth and length of time in the United States in 4 categories: 1) foreign born and lived in the United States less than 10 years (score = 0), 2) foreign born and lived in the United States 10 to 19 years (score = 1), 3) foreign born and lived in the United States for 20 years or more (score = 2), and 4) born in the United States (score = 3). A score of 0 to 1 was assigned on the basis of language spoken at home in 2 categories: 1) native language (score = 0), or 2) English only or English and other language(s) (score = 1). These scores were summed to a total acculturation score, ranging from 0 (least acculturated) to 5 (most acculturated) and were validated in a sample of Hispanic and Chinese women (25).

**Other covariates**. Covariates considered were maternal age, mother’s marital status when the baby was born (married vs unmarried), mother’s educational attainment (less than a high school diploma, high school diploma, some college, college degree or above), and annual household income (<$20,000, $20,000–$39,999, $40,000–$59,999, $60,000–$99,999, ≥$100,000, “I don't know”), stressful life events (measured by trauma stressor score, family stressor score, financial stressor score, and emotional stressor score), prepregnancy BMI status (we used the BMI ranges for Asians: normal weight, BMI of 18.5–22.9; overweight, BMI of 23.0–27.4; and obese, BMI ≥27.5 [27]), exercise more than 30 minutes per day during the last 3 months of pregnancy (no days, exercised <1 day, exercised 1–4 days, exercised ≥5 days, or told by a doctor not to exercise), and the Kessner Index of adequacy in prenatal care (adequate, intermediate, or inadequate). The Kessner Index was calculated on the basis of the month of pregnancy in which prenatal care began, number of total prenatal care visits, and gestational age at delivery. The algorithm for this calculation was published previously (26).

### Statistical analysis

We reported both crude and weighted gestational diabetes prevalence on the basis of the complex sampling design in the LAMB study. We presented weighted descriptive results as mean (standard error [SE]) for continuous variables or percentage for categorical variables by racial/ethnic group.

We applied multivariate, weighted logistic regressions to estimate the adjusted odds ratio (aOR) and 95% confidence interval (CIs) for comparing gestational diabetes prevalence among racial/ethnic groups and subgroups of Asian women, adjusting for known risk factors for gestational diabetes (ie, maternal age, education, marital status, income, prepregnancy BMI, adequacy of prenatal care, physical activity, stress during pregnancy, and neighborhood contextual factors). We did not conduct multivariate regressions comparing subgroups of Asian women because of the insufficient sample size in each subgroup (eg, we had fewer than 30 Japanese women). Finally, as a sensitivity analysis we used the Asian-specific BMI-cutoff threshold to define the BMI status among Asian subgroups, which did not notably change our results in the multivariate analyses.

We conducted a mediation analysis using STATA’s medeff module (STATACorp LLC) to test the extent to which the difference in prevalence across different racial/ethnic groups can be explained by acculturation, setting up the acculturation score as the mediator variable between the Asian race variable and the gestational diabetes outcome. Significance was set at *P* <.05 (2 tailed). Finally, as a sensitivity analysis we used the Asian-specific BMI-cutoff threshold to define the BMI status among the Asian subsample to see if changing the BMI-cutoff points for Asians notably changed our results in the multivariate analyses.

## Results

Of the 5,562 women included in this study, 21.9% (n = 1,216) were non-Hispanic white, 15.9% (n = 883) were non-Hispanic black, 44.8% (n = 2,492) were Hispanic, and 16.0% (n = 890) were Asian. Overall, 588 (10.6%) women had gestational diabetes during their pregnancy. The weighted prevalence was 7.9% among non-Hispanic white women, 10.7% among non-Hispanic black women, 9.0% among Hispanic women, and 15.5% among Asian women overall (Figure). Among Asian subgroups, the weighted prevalence of gestational diabetes was 7.9% among Japanese women, 9.2% among Korean women, 15.0% among Filipina women, 17.3% among Chinese women, 21.2% among Vietnamese women, and 24.9% among Asian Indian women.

**Figure:**
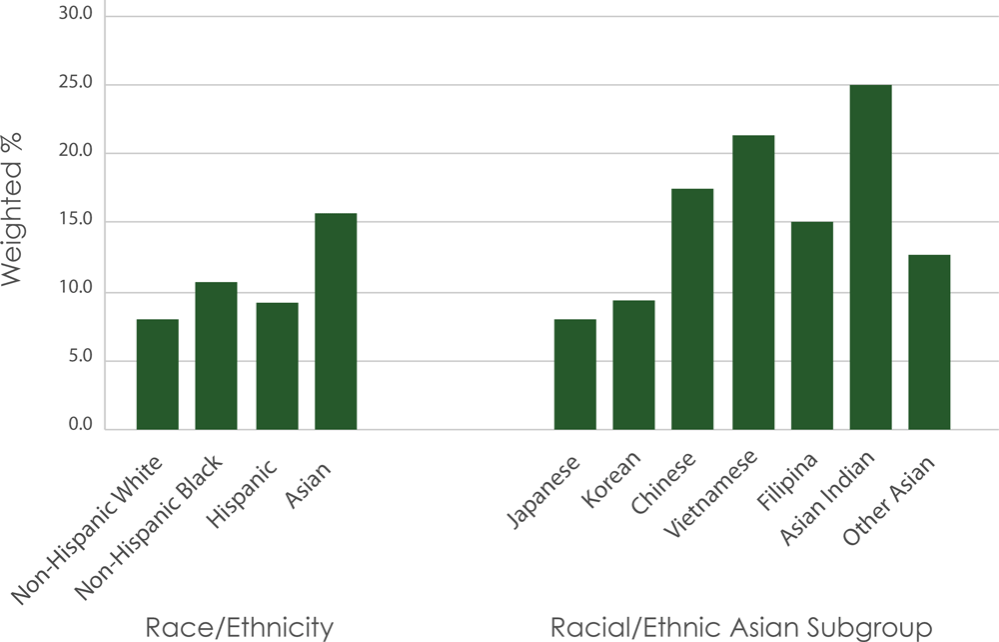
Weighted prevalence of gestational diabetes mellitus among participants (N = 5,562), by racial/ethnic group, Los Angeles Mommy and Baby Study, 2007. *P* value <. 05 as compared with the reference group, non-Hispanic white women.

**Table:** 

Race/Ethnicity	Weighted %
Non-Hispanic white	7.9
Non-Hispanic black	10.7
Hispanic	9.0
Asian	15.5
Asian subgroups	
Japanese	7.9
Korean	9.2
Chinese	17.3
Vietnamese	21.2
Filipino	15.0
Indian	24.9
Other Asian	12.7

Maternal age, marital status when baby born, education, household income, prepregnancy BMI, and physical activity during pregnancy were associated with gestational diabetes and differed across racial/ethnic groups, whereas stress during pregnancy and adequacy of prenatal care were related only to racial/ethnic groups, not gestational diabetes (Table 1). On average, Asian and non-Hispanic white women in our sample did not differ statistically in maternal age, education, income, adequacy of prenatal care, and physical exercise level; however, Asian women were more likely to be married at the baby’s birth (*P* < .001) and had lower prepregnancy BMI than non-Hispanic white women (*P* < .001). Asian women also had the lowest acculturation score across all racial/ethnic groups.

**Table 1:** Characteristics of Participants Without Pre-Existing Diabetes (N = 5,562), by Race/Ethnicity and Gestational Diabetes Status, Los Angeles County Mommy and Baby Study 2007^a^

Characteristic	Race/Ethnicity^b^					Gestational Diabetes		
	Non-Hispanic White (N = 1,216)	Non-Hispanic Black (N = 883)	Hispanic (N = 2,492)	Asian (N = 890)	*P *Value^c^	Yes (N = 588)	No (N = 4,974)	*P *Value^d^
**Maternal age, y, weighted mean (SE)**	30.88 (5.99)	27.60 (6.39)	27.04 (6.21)	30.82 (5.36)	<.001	31.33 (5.87)	28.82 (6.22)	<.001
**Maternal age group, y, **								
<18	0.48	3.05	3.80	0.98	<.001	0.52	2.67	<.001
18–24	13.77	29.74	31.66	10.21		12.41	25.80	
25–34	59.24	53.05	49.77	59.37		43.97	50.47	
35–44	32.01	14.05	14.77	19.89		32.97	18.98	
≥45	0.24	0.11	0.00	0.11		0.17	0.28	
**Married when baby born, %**	78.82	35.19	49.39	83.72	<.001	65.75	59.58	<.001
**Educational attainment, %**								
Less than high school diploma	5.10	12.12	33.80	3.33	<.001	21.92	17.89	.007
High school diploma	14.55	27.02	33.21	12.53		22.73	24.49	
Some college	27.57	39.95	22.73	26.55		25.49	27.67	
College degree or above	52.77	20.90	10.25	57.59		29.87	29.96	
**Household income, %**								
<$20,000	15.08	46.03	51.86	16.27	<.001	33.20	40.91	.006
$20,000–$39,999	13.37	22.48	25.16	19.85		25.18	21.88	
$40,000–$59,999	11.79	10.29	7.35	15.23		11.97	9.11	
$60,000–$99,999	24.37	9.92	6.49	24.38		13.95	12.12	
≥$100,000	32.60	8.79	3.28	22.68		11.56	11.45	
I don't know	2.79	2.49	5.86	1.58		4.14	4.53	
**BMI prepregnancy, weighted mean (SE)**	24.20 (4.94)	26.25 (6.22)	26.14 (5.85)	22.15 (4.11)	<.001	26.99	24.96	<.001
**BMI status (weight in kg/height in m^2^), %**								
Normal weight (<25)	4.75	6.01	14.06	16.21	<.001	45.45	62.53	<.001
Overweight (25–29.9)	62.18	45.92	42.26	66.63		25.65	22.84	
Obese (≥30)	21.84	26.39	23.91	11.26		28.90	15.62	
**Stress during pregnancy, weighted mean stressor score (SE)**								
Trauma	0.29 (0.58)	0.41 (0.65)	0.31 (0.58)	0.23 (0.54)	<.001	0.31 (0.02)	0.31 (0.01)	.98
Family	0.45 (0.82)	0.92 (1.07)	0.58 (0.89)	0.35 (0.68)		0.56 (0.03)	0.57 (0.01)	.71
Financial	0.30 (0.63)	0.66 (0.84)	0.50 (0.78)	0.26 (0.59)		0.49 (0.03)	0.44 (0.01)	.07
Emotional	0.24 (0.49)	0.46 (0.68)	0.32 (0.55)	0.23 (0.47)		0.30 (0.02)	0.31 (0.01)	.61
**Kessner Index^e^**								
Inadequate	0.85	2.30	2.36	1.25	<.001	1.76	1.87	.24
Intermediate	5.13	10.86	11.32	6.04		8.42	9.20	
Adequate	86.71	82.20	76.51	87.90		86.24	82.96	
Unavailable	7.31	4.64	9.81	4.81		3.58	5.97	
**Acculturation score^f^, weighted mean (SE)**	3.60 (0.99)	3.74 (0.84)	2.52 (1.58)	1.98 (1.49)	<.001	2.61	2.93	<.001
**Days of exercise >30 min, %**								
<1	38.96	37.34	35.14	26.78	<.001	21.05	24.25	.01
1–4	41.14	44.00	42.37	43.68		53.91	56.93	
≥5	10.65	13.33	16.41	12.64		15.79	12.92	
Told not to exercise	9.02	5.33	6.08	4.74		9.26	5.90	

Abbreviations: BMI, body mass index; SE, standard error.

a The 2007 Los Angeles Mommy and Baby Study: a multilevel, population-based study of maternal and infant health in Los Angeles County (24).

b Numbers do not total 5,562 because 81 women fell in an “other” race/ethnicity category. We do not report descriptive statistics for them because of potentially unstable estimates from small counts of the cells.

c *P* values are for comparison across racial/ethnic groups.

d *P* values are for comparison between women with and women without gestational diabetes.

e The Kessner Index was calculated on the basis of the month of pregnancy in which prenatal care began, number of total prenatal care visits, and gestational age at delivery. The algorithm for this calculation was published previously (26).

f The acculturation score was calculated on the basis of participants’ responses to 3 questions assessing establishing country of birth, language spoken at home, and length of time in the United States. A 0 to 3 score was assigned on the basis of country of birth and length of time in the United States in 4 categories: 1) foreign born and lived in the United States less than 10 years (score = 0), 2) foreign born and lived in the United States for 10 to 19 years (score = 1), 3) foreign born and lived in the United States for 20 years or more (score = 2), and 4) born in the United States (score = 3). A score of 0 to 1 was assigned on the basis of language spoken at home in 2 categories: 1) native language (score = 0), or 2) English only or English and other language(s) (score = 1). These scores were summed to a total acculturation score, ranging from 0 (least acculturated) to 5 (most acculturated) (25).

Compared with non-Hispanic white women, the aOR of having gestational diabetes was 1.28 among non-Hispanic black women (95% CI, 0.90–1.82; *P* = .17), 1.45 among Hispanic women (95% CI, 1.07–1.95; *P* = .02), and 2.44 among Asian women (95% CI, 1.81–3.29; *P* < .001) after adjusting for all other covariates (Table 2). After additionally adjusting for acculturation, the aORs among Hispanic women (aOR = 1.32; 95% CI, 0.96–1.80; *P* = .09) and Asian women (aOR = 2.08; 95% CI, 1.50–2.87; *P* < .001) were both attenuated, but remained significant among Asian women (Table 2,). The acculturation score was negatively associated with having gestational diabetes (aOR = 0.93; 95% CI, 0.86–0.99; *P* = .03). Mediation analysis showed that 15.9% (95% CI, 11.4%–25.1%; *P* < .001) of the association between race and gestational diabetes was explained by the acculturation score.

**Table 2:** Prevalence of Gestational Diabetes Mellitus Among Participants (N = 5,562), by Race/Ethnicity, With and Without Adjusting for Acculturation Score, Los Angeles County Mommy and Baby Study, 2007^a^

Variable	Multivariate Model 1^b^		Multivariate Model 2^c^		% Total Effect Mediated Through Acculturation (95% CI)
	aOR (95% CI)	*P* Value	aOR (95% CI)	*P* Value	
Non-Hispanic white	1 [Reference]		1 [Reference]		15.9 (11.4–25.1)
Non-Hispanic black	1.28 (0.90–1.82)	.17	1.29 (0.91–1.83)	.15	
Hispanic	1.45 (1.07–1.95)	.02	1.32 (0.96–1.80)	.09	
Asian	2.44 (1.81–3.29)	<.001	2.08 (1.50–2.87)	<.001	
Acculturation score^d^	NA	NA	0.93 (0.86–0.99)	.03	

Abbreviations: CI, confidence interval; NA, not applicable; aOR, adjusted odds ratio.

a The 2007 Los Angeles Mommy and Baby Study: a Multilevel, Population-Based Study of Maternal and Infant Health in Los Angeles County (24).

b Model 1: adjusted for maternal age (continuous), marital status when baby born (married vs unmarried), 2006 annual household income (<$20,000, $20,000–$39,999, $40,000–$59,999, $60,000–$99,999, ≥$100,000, or unknown), mother’s education (less than high school diploma, high school diploma, some college, college degree or above), Kessner Index for adequacy of prenatal care (adequate, intermediate, or inadequate), trauma stressor score, family stressor score, financial stressor score, emotional stressor score, days of more than 30 minutes of exercise (none, <1, 1–4, ≥5, or told by the doctor to exercise), prepregnancy body mass index (normal, overweight, or obese).

c Model 2: model 1 plus acculturation score. Model 2 examines the percentage of the association between Asian race and gestational diabetes that was mediated through acculturation, with the same list of covariates used in Model 1.

d The acculturation score was calculated on the basis of participants’ responses to 3 questions assessing country of birth, language spoken at home, and length of time in the United States. A 0 to 3 score was assigned on the basis of country of birth and length of time in the United States in 4 categories: 1) foreign born and lived in the United States less than 10 years (score = 0), 2) foreign born and lived in the United States for 10 to 19 years (score = 1), 3) foreign born and lived in the United States for 20 years or more (score = 2), and 4) born in the United States (score = 3). A score of 0 to 1 was assigned on the basis of language spoken at home in 2 categories: 1) native language (score = 0), or 2) English only or English and other language(s) (score = 1). These scores were summed to a total acculturation score, ranging from 0 (least acculturated) to 5 (most acculturated) (25).

As a sensitivity analysis we used the Asian-specific BMI-cutoff threshold to define the BMI status among the Asian subsample and found that changing the BMI-cutoff points for Asians did not notably change our results in the multivariate analyses.

## Discussion

In this large multiple-race, population-representative study of 5,562 women in Los Angeles County, we found that Asian women had the highest gestational diabetes prevalence (15.5%) among all racial/ethnic groups. The odds of having gestational diabetes were about 2.44 times higher among Asian women than among non-Hispanic white women, independent of known risk factors (eg, maternal age, education, marital status, income, exercise, stress, adequacy of prenatal care, geographic effect). Acculturation was negatively associated with gestational diabetes and mediated the association between race/ethnicity and gestational diabetes. To our knowledge, this is the first study that used a population-representative sample with measures of demographic, lifestyle, behavioral, psychosocial, and cultural factors.

The potential mechanisms by which Asian women have a higher risk of developing gestational diabetes are not well understood and likely involve multiple factors, including genetic, lifestyle, social–cultural, and other environmental factors. Prepregnancy obesity is a known strong risk factor for gestational diabetes. However, Asian women have lower BMIs than women of other racial/ethnic groups (28,29). We confirmed that Asian American women in our study overall had a lower BMI than women of other racial/ethnic groups and were less likely to be overweight or obese. In a large US-based study, 76.8% of African-American women, 59.8% of Hispanic women, and 46.2% of white women had a BMI greater than 25 compared with only 24.9% of Asian women (28). In another US study, the adjusted population attributable fraction of gestational diabetes to overweight and obesity (BMI ≥25) was 38.6% overall, 41.2% among non-Hispanic white women, 44.2% among Hispanic women, 51.2% among non-Hispanic black women, and only 17.8% among Asian women (29). In our study, we found that Asian women had a rate of gestational diabetes 2.44 times higher than non-Hispanic white women after controlling for prepregnancy BMI, suggesting that other factors must also play roles in the relationship between Asian race/ethnicity and gestational diabetes.

Recent studies suggested that culture and socioeconomic factors may partially explain the observed racial/ethnic difference of gestational diabetes risk. Other things being equal, immigrants are more likely to be socially disadvantaged and have lower income than those born locally, with potential effects on their overall health (30,31). In our study, the proportion of Asian women with an annual income below $40,000 was higher than among non-Hispanic white women but lower than among non-Hispanic black and Hispanic women. However, after we controlled for income in our multiple regression models, the gestational diabetes risk remained higher among Asian women than among white women, suggesting that income status alone would not explain the higher gestational diabetes prevalence among Asian women.

The acculturation score — a scale based on whether born in the United States, length of residency in the United States, and language spoken at home — had a significant negative association with gestational diabetes risk in our study. Previous studies found a mixed effect of acculturation on health outcomes. Higher acculturation has been associated with increased risk of diabetes among Hispanic people in a US study (21) and among Chinese people in an Australia study (22), probably because higher acculturation is associated with worsening diet quality (20,32) and increased stress (eg, separation from family, discrimination) (33). Studies comparing foreign-born women and US-born women across different races/ethnicities found that being born outside of the United States was associated with an increased risk of gestational diabetes among black, Asian Indian, Chinese, and Filipina women but decreased risk among Japanese and Korean women (13,14). Dietary factors have also been identified as risk factors for gestational diabetes, independent of prepregnancy BMI and other known risk factors (34). Acculturation is related to nutrition, lifestyle, and use of health services (35,36). Thus, acculturation may be a proxy measure for a combination of culture and dietary factors and other lifestyle changes for health outcomes (37). Unfortunately, we did not have dietary intake data in our study. We are not aware of published studies that directly examined the relationship between acculturation and gestational diabetes. Thus, this negative association between acculturation and gestational diabetes, independent of other risk factors, is novel. Future studies can further examine whether diet or other lifestyle factors play important roles in the excess gestational diabetes risk among Asian women in the United States. Meanwhile, we are also aware that the association between Asian race and gestational diabetes remained significant (though slightly attenuated) after we controlled for acculturation. Results from the mediation analysis suggested acculturation explained about one-sixth (15.9%) of the observed association between being Asian and gestational diabetes risk, meaning that acculturation alone still does not fully explain the excess gestational diabetes risk among Asian women.

This study had several strengths. We used a large sample size with a population-representative sample and measures of socioeconomic, behavioral, lifestyle, and psychological factors. It also had limitations. One limitation was that gestational diabetes was self-reported. The LAMB study did not have information on the blood glucose levels of participants. However, the self-reported measure of gestational diabetes showed a high validity when compared with a physician diagnosis in the Nurses’ Health Study II (38), probably because women with gestational diabetes would be referred for glucose monitoring and receive lifestyle consultation and medical treatment from their providers. Another limitation was that we did not have dietary intake information in the LAMB study to allow us to assess the association between diet and gestational diabetes risk.

Our findings confirmed that Asian women had the highest gestational diabetes prevalence among all racial/ethnic groups. Acculturation was negatively associated with gestational diabetes and partially explained the race–gestational diabetes association. Clinicians should be aware of the high gestational diabetes risk in Asian women and provide screening at their first prenatal care visit as recommended by the American Diabetes Association (39). Further studies with more detailed information on dietary intake and body fat distribution are warranted to explore the underlying mechanisms by which Asian women have an increased risk of developing gestational diabetes.
